# Big five personality traits and employability: The mediating role of internship attitudes among chinese vocational students

**DOI:** 10.1371/journal.pone.0329103

**Published:** 2025-08-05

**Authors:** Sixiao Hu, Mohd Khata Jabor, Hongbin Fang, Dapeng Sun, Yiqing Zheng, Yunzhuo Wu, Yuefan Zhao, Yuanyuan Zhao, Ninglin Li, Xiaohong Zhao

**Affiliations:** 1 Department of Information Engineering, Hebei Institute of Mechanical and Electrical Technology, Xingtai, China; 2 Faculty of Educational Sciences and Technology, Universiti Teknologi Malaysia, Johor Bahru, Malaysia; 3 Department of Student Affairs Management, Hebei Institute of Mechanical and Electrical Technology, Xingtai, China; 4 School of Marxism, Hebei Institute of Mechanical and Electrical Technology, Xingtai, China; 5 Mechnical and Eletrical Engineering Institute, Xingtai University, Hebei Institute of Mechanical and Electrical Technology, Xingtai, China; 6 Department of Mechanical Engineering, Hebei Institute of Mechanical and Electrical Technology, Xingtai, China; 7 Department of Materials and Building Engineering, Hebei Institute of Mechanical and Electrical Technology, Xingtai, China; 8 Faculty of Management, Universiti Teknologi Malaysia, Johor Bahru, Malaysia; 9 Tokyo University of the Arts, Graduate School of Fine Arts, Japan; Ho Chi Minh City University of Medicine and Pharmacy, VIETNAM

## Abstract

This study investigates the impact of the Big Five personality traits on the employability of vocational education students, with a focus on the mediating role of internship attitudes. Data were collected from 550 students enrolled in 11 vocational colleges in Hebei Province, China, using a structured questionnaire. Structural Equation Modeling (SEM) was employed to analyze the relationships among personality traits, internship attitudes, and employability. The findings reveal that personality traits significantly influence internship attitudes, which in turn affect employability. These results provide novel theoretical insights into the psychological mechanisms underlying internship persistence and practical recommendations for optimizing vocational education and enhancing school-enterprise collaboration to better meet students’ employability and career readiness needs.

## Introduction

The global employment landscape is undergoing profound transformations driven by technological advancements and the Fourth Industrial Revolution, encompassing artificial intelligence and robotics [1]. According to the Future of Jobs Report, over 85% of companies are projected to accelerate technology adoption by 2025, with 40% of workers requiring reskilling to meet evolving job demands. Vocational education has emerged as a pivotal solution to address global skill mismatches [2]. For instance, Germany’s dual education system achieves this by fostering close collaboration between enterprises and schools, equipping apprentices with practical experience and vocational skills [3]. Similarly, Japan’s work-integrated learning model emphasizes the integration of technical applications and academic knowledge through embedded workplace training. These successful models offer valuable insights for global vocational education reforms [4].

Despite its scale and market-oriented approach, China’s vocational education system faces significant challenges, particularly in enhancing school-enterprise collaboration and cultivating students’ career readiness [5]. As the world’s largest vocational education system, China’s vocational colleges play a critical role in developing skilled talent [6]. By 2023, vocational colleges enrolled over 11 million students, accounting for 38% of all higher education students. Despite an initial employment rate of 86.6%, approximately 42% of employers report that graduates’ professional skills fail to meet job requirements [7]. This highlights an urgent need to enhance the employability of vocational students and optimize vocational education systems to align with the rapidly evolving labor market.

Vocational students face distinct challenges in career preparation compared to university students, particularly in developing career awareness and fostering positive internship attitudes [8]. Internships, defined as structured opportunities for students to apply theoretical knowledge in real-world contexts, are a cornerstone of vocational education. However, the effectiveness of internships largely depends on students’ attitudes, encompassing their perceptions of the relevance, value, and expected outcomes of these experiences [9]. Positive internship attitudes are strongly associated with higher engagement, improved skill acquisition, and enhanced career readiness [10].

Simultaneously, individual personality traits play a critical role in shaping these attitudes. The Big Five personality traits framework—comprising conscientiousness, extraversion, agreeableness, openness, and neuroticism—is widely recognized as a robust model for understanding personality differences and their behavioral implications [11]. These traits have consistently been linked to various career outcomes, including engagement, performance, and adaptability [12]. For example, Feng et al. [13] demonstrated that conscientiousness and openness strongly predict vocational engagement, while Gao et al. [14] highlighted the mediating role of internship attitudes in skill development. However, research exploring the interplay between personality traits and internship attitudes among vocational students remains scarce [15]. Furthermore, the concept of employability—defined as the combination of skills, knowledge, and attributes that enable individuals to secure and sustain employment has received limited attention in vocational education contexts, particularly regarding how personality traits and internship attitudes collectively shape employability [16].

This study addresses these gaps by systematically examining the associations among the Big Five personality traits, internship attitudes, and employability, with a specific focus on the mediating role of internship attitudes [17]. By positioning internship attitudes as a key variable, this study extends the application of the Big Five personality framework in vocational education and uncovers the mechanisms through which personality traits influence employability [18]. Unlike prior studies that primarily focus on the direct effects of personality traits on career outcomes, this research emphasizes the bridging role of internship attitudes, offering a novel theoretical framework that deepens the understanding of employability within vocational education contexts [19].

The study draws on survey data from 550 vocational college students in China and employs Structural Equation Modeling (SEM) to empirically analyze the associations among the Big Five personality traits, internship attitudes, and employability [20]. This approach provides systematic insights into the mechanisms and pathways through which these variables interact [21].

The findings of this study carry significant implications for educators, policymakers, and industry practitioners [22]. In China, these results can guide vocational colleges in designing internship programs that align with students’ personality traits, foster positive attitudes, and improve employability [23]. Internationally, the theoretical framework provides valuable insights into addressing skill mismatches and advancing personalized vocational education strategies, particularly in developing economies [24]. This research contributes to global vocational education reforms by demonstrating how the integration of personality traits and attitudes can enhance career readiness, offering actionable recommendations for stakeholders in the education sector [25].

## Research questions

- How do the Big Five personality traits influence the employability of vocational college students?
- What is the mediating role of internship attitudes in the association between the Big Five personality traits and employability?
- How do the Big Five personality traits shape internship attitudes?

## Literature review

### Employability and its significance

Employability is a critical measure of an individual’s competitiveness and adaptability in the job market [26]. In today’s rapidly evolving workplace, understanding the factors influencing employability is essential for effective education and career guidance [27]. Defined as the combination of skills, knowledge, and attributes that enable individuals to secure and sustain employment, employability extends beyond technical competencies to encompass personal and behavioral traits [27]. In this context, vocational education plays a vital role in equipping students with both technical and adaptive skills required to meet the dynamic demands of industries [28]. Enhancing employability among vocational students requires a comprehensive understanding of the psychological and experiential factors that contribute to their career readiness [29].

### Big Five personality traits and employability

The Big Five personality traits framework—comprising conscientiousness, extraversion, agreeableness, openness, and neuroticism—is widely recognized as a robust model for understanding personality differences and their behavioral implications [30]. Conscientiousness is characterized by traits such as being organized, responsible, and goal-oriented, which are likely to enhance students’ ability to complete tasks effectively during internships and improve their employability [31]. Extraversion involves being sociable, assertive, and energetic, which may positively influence students’ networking and leadership skills. Agreeableness reflects a person’s cooperativeness, empathy, and trustworthiness, contributing to better teamwork and interpersonal associations [32]. Openness to experience indicates a willingness to try new things, curiosity, and creativity, which can facilitate adaptation to new work environments and tasks. Neuroticism, on the other hand, is associated with emotional instability and anxiety, which may hinder students’ performance and stress management during internships. Further research could explore these associations in more detail to enhance the theoretical understanding of how each personality trait influences internship behavior and employability [33].

These findings highlight the multifaceted ways in which personality traits influence employability, underscoring the importance of tailoring vocational education programs to leverage individual personality strengths while addressing potential weaknesses [34].

### Internship attitudes and employability

Internship attitudes—students’ perceptions of the relevance, value, and expected outcomes of their practical experiences—play a pivotal role in shaping employability. Positive internship attitudes enhance career adaptability and skill acquisition, empowering students to transition from theoretical learning to workplace application [35]. Studies indicate that students with strong learning orientation and high engagement during internships are better positioned to develop both technical and soft skills, key components of employability [36]. Furthermore, internship attitudes mediate the effects of personality traits on employability, amplifying the positive influence of traits like conscientiousness and openness while mitigating the negative effects of neuroticism [37]. This mediating role underscores the importance of fostering positive internship experiences to bridge the gap between education and employability [38].

### Big Five personality traits and internship attitudes

The interplay between personality traits and internship attitudes has gained increasing attention in recent research, highlighting the indirect role of personality traits in employability through their influence on internship attitudes. Students with high extraversion demonstrate proactivity and engagement during internships, leveraging social interactions for greater satisfaction and success [39]. Agreeable students excel at building harmonious associations, facilitating constructive feedback and guidance from mentors [40]. Conscientious students exhibit strong task orientation and goal-driven behaviors, leading to higher performance and practical skill acquisition. Openness promotes innovative thinking and adaptability, enriching students’ learning experiences during internships [41]. Conversely, high neuroticism often results in lower engagement and performance due to emotional instability and stress intolerance [42].

By influencing students’ attitudes and behaviors, personality traits indirectly shape their internship outcomes, which in turn impact their employability. These findings emphasize the importance of integrating personality-focused strategies into vocational education and internship programs [43].

### The mediating role of internship attitudes

While existing research has explored the direct effects of personality traits on employability, the mechanisms through which these traits influence employability remain underexplored [44]. Internship attitudes act as a critical mediator, bridging personality traits and employability by transforming students’ behavioral tendencies into practical career outcomes [45]. For instance, Shinnar et al. [46] found that conscientiousness and openness indirectly enhance employability through improved engagement and learning orientation during internships. This mediating role highlights the need for holistic approaches in vocational education, integrating personality development with structured internship experiences to enhance employability [47].

### Conceptual model and hypothesis development

**Fig 1:**
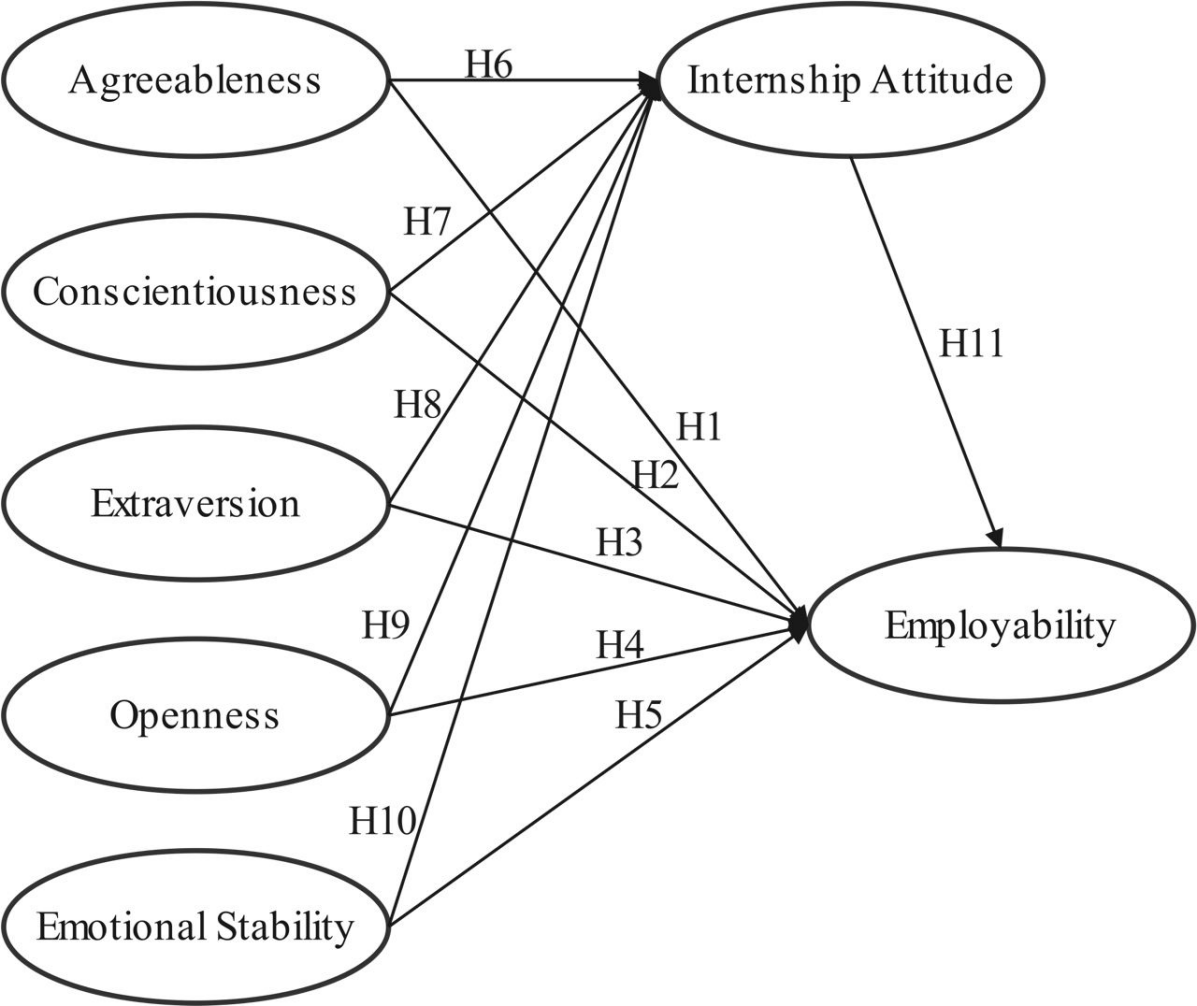
Conceptual framework.

Drawing on an extensive body of literature and foundational theories, including the Big Five Personality Traits Theory [48], the Theory of Planned Behavior (Ajzen, 1985), and Human Capital Theory (Becker, 1964), a conceptual research model was constructed to examine the associations among key variables (see Fig 1). These theories collectively provide a robust framework to explore how Big Five Personality Traits are associated with employability through the mediating role of internship attitudes, offering insights into the mechanisms that shape career outcomes for Chinese business students [49].

Supplementary Notes:

The model illustrates the direct path associations between Big Five Personality Traits (BFPT), Internship Attitude (IA), and Employability (EMP) (H1-H11). The mediation hypotheses (H12-H16) are not directly presented in the diagram but are supplemented below, highlighting the mediating role of Internship Attitude (IA) in the associations:

Mediation Hypotheses:

H12: Internship Attitude (IA) mediates the association between Extraversion and Employability (EMP).

H13: Internship Attitude (IA) mediates the association between Agreeableness and Employability (EMP).

H14: Internship Attitude (IA) mediates the association between Conscientiousness and Employability (EMP).

H15: Internship Attitude (IA) mediates the association between Emotional Stability (Neuroticism) and Employability (EMP).

H16: Internship Attitude (IA) mediates the association between Openness and Employability (EMP).

By integrating the Big Five Personality Traits Theory, the Theory of Planned Behavior (TPB), and Human Capital Theory (HCT), this study aims to construct a comprehensive framework that explores the interplay between personality traits, internship attitudes, and employability [50]. Specifically, the framework examines how openness, conscientiousness, extraversion, agreeableness, and neuroticism are associated with internship attitudes and how these attitudes, in turn, mediate the association between personality traits and employability [51]. This framework also considers contextual factors relevant to Chinese business students, including cultural influences and the unique challenges of transitioning from education to the workforce [52]. Based on this theoretical foundation, the study proposes the following hypotheses to investigate these associations systematically [53].

**Hypothesis 1 (H1):** There is a significant positive association between extraversion and employability.

**Hypothesis 2 (H2):** There is a significant positive association between agreeableness and Employability.

**Hypothesis3(H3):** There is a significant positive association between conscientiousness and Employability.

**Hypothesis 4 (H4):** There is a significant negative association between neuroticism and Employability.

**Hypothesis 5 (H5):** There is a significant positive association between openness to experience and Employability.

**Hypothesis 6 (H6):** There is a significant negative association between extroversion and internship attitude.

**Hypothesis 7 (H7):** There is a significant positive association between agreeableness。and internship attitude.

**Hypothesis8(H8):** There is a significant positive association between conscientiousness and internship attitude.

**Hypothesis 9(H9):** There is a significant negative association between neuroticism and internship attitude.

**Hypothesis 10(H10):** There is a significant negative association between openness to experience and internship attitude.

**Hypothesis11(H11):** There is a significant positive association between Internship attitude and Employability.

**Hypothesis12(H12):** Internship attitude mediates the association between。extraversion and employability.

**Hypothesis13(H13):** Internship attitude plays a mediating role in the association between agreeableness and internship intention.

**Hypothesis14(H14):** Internship attitude mediates the association between consciousness and internship intention.

**Hypothesis15(H15):** Internship attitude mediates the association between neuroticism and internship intention.

**Hypothesis 16 (H16):** Internship attitude mediates the association between openness。 to experience and continued internship intention.

## Research methodology

Structural Equation Modeling (SEM) using AMOS 27 software was applied to analyze the associations among the Big Five personality traits, internship attitudes, and students’ persistence intentions. While SEM is a powerful tool for evaluating theoretical models and testing associations among latent constructs, it is important to note that SEM does not establish causal associations, especially in cross-sectional research designs. Instead, it provides correlational evidence to support theory-driven hypotheses.

AMOS 27 was chosen for this analysis due to its robust capabilities in handling complex associations between latent variables and observed variables [54]. It provides a comprehensive set of fit indices that allow for rigorous evaluation of the measurement and structural models [55]. Additionally, AMOS offers user-friendly interfaces and extensive documentation, facilitating accurate model specification and interpretation of results. Compared to other statistical methods, SEM in AMOS allows for the simultaneous estimation of measurement errors and the associations among constructs, providing more reliable and valid results [56].

### Participant recruitment

Participant recruitment was conducted between 01/11/2024 and 31/01/2025. A total of 550 accounting students from 11 vocational colleges in Hebei Province, China, were invited to participate. Recruitment was carried out using a combination of email invitations and in-person sessions at the students’ respective institutions.

### Data collection

Quantitative data were collected using a structured questionnaire. All participants were provided with detailed information about the study, including its objectives, procedures, and potential risks, prior to their involvement. Participants were also informed of their rights, such as the ability to withdraw from the study at any time without consequences.

All participants provided informed consent, documented in either written or verbal form based on their preference. Verbal consent was further supported by recordings or written confirmation from research staff. The study exclusively involved participants aged 18 years or older, with no minors included.

### Ethical approval and informed consent

This study received ethical approval from the UTM Research Ethics Committee (Approval No. UTM20241018). Participant recruitment and data collection were conducted between 01/11/2024 and 31/01/2025, strictly adhering to the approved research timeline and ethical principles. These principles included maintaining participant confidentiality, obtaining informed consent, and ensuring the right to withdraw from the study at any time without penalty.

All participants were provided with detailed information about the study’s objectives, procedures, and potential risks before their involvement. Informed consent was obtained from all participants in either written or verbal form, depending on their preference. Written consent was documented through signed consent forms, while verbal consent was recorded and confirmed by the research team to ensure proper documentation and compliance with ethical standards.

The study exclusively involved participants aged 18 years or older, with no minors included. Therefore, consent from parents or guardians was not applicable. The UTM Research Ethics Committee did not waive the need for informed consent.

The original ethics approval document is in English and has been provided as a supplementary file.

### Supporting information

The original ethics approval document (in Chinese) and its English translation are provided as supporting materials to demonstrate the study’s adherence to ethical standards and approval requirements.

### Questionnaire design

A survey (questionnaire) is used to measure observed variables including observed variables Big Five Personality Traits (Extraversion, Agreeableness, Conscientiousness, Emotional Stability, Openness) [57], Internship Attitude (IA), and Employability. The employability construct includes subdimensions such as Soft Skills (SS), General Business Requirements (GBR), Audit and Assurance (AA), Conceptual Knowledge (CK), and Special Topics in Accounting (STA) [58]. Each variable is measured using multiple items adapted from validated scales to ensure reliability and validity. The questionnaire comprises 80 items rated on a five-point Likert scale and is designed to capture the associations between personality traits, attitudes, and employability outcomes among vocational students [59]. The survey was originally developed in English and translated into Chinese to suit the cultural and linguistic context of respondents from Sichuan and Chongqing vocational colleges in China [60].

### Instrument development

The Big Five Personality Traits were measured using the NEO-PI-R scale developed by McCrae and Costa, which includes five dimensions: extraversion, agreeableness, conscientiousness, emotional stability, and openness. These dimensions assess individuals’ psychological tendencies and behavioral characteristics [61]. The reliability coefficient of the Big Five Personality Traits scale was 0.951, indicating high reliability.

Internship Attitudes were measured using a multidimensional approach based on Bates et al. (2019), consisting of 8 items covering three dimensions: learning orientation, engagement, and career adaptability. The reliability of the internship attitudes scale was also very high, with a Cronbach’s alpha value of 0.944.

To assess respondents’ perceptions of employability, a framework integrating soft skills, technical skills, conceptual knowledge, and general business requirements was used [62]. The framework, adapted from Yorke (2006), included 29 items and was tailored to align with the practical context of vocational education in China, ensuring the reliability of the variable. The reliability coefficient of the employability scale was 0.946, confirming the robustness of the measurement [63].

To evaluate the reliability of this survey instrument, a pilot study was conducted with 30 respondents using a 5-point Likert scale (ranging from 1 for “strongly agree” to 5 for “strongly disagree”). Following the guidelines of Hair, Hult, Ringle, and Sarstedt (2013) and Nunnally and Bernstein (1994), a reliability score of.70 or above is deemed acceptable. This survey achieved a Cronbach’s alpha of.958, indicating a high level of reliability. In addition, exploratory factor analysis (EFA) was also conducted to group observed variables into unobserved second order constructs. The questionnaire is thoughtfully organized into first order and second-order dimensions to thoroughly capture the intricacies and depths of the measured variables (see Table 3).

**Table 3:** Normality test of internship attitude.

Variable name	Sample size	Minimum value	Maximum value	Mean value	Standard deviation	skewness	kurtosis
IA1	381	1	5	2.38	1.098	0.595	−0.256
IA2	381	1	5	2.32	1.089	0.516	−0.416
IA3	381	1	5	2.35	1.092	0.524	−0.404
IA4	381	1	5	2.36	1.078	0.646	−0.034
IA5	381	1	5	2.42	1.094	0.496	−0.33
IA6	381	1	5	2.41	1.036	0.515	−0.168
IA7	381	1	5	2.38	1.068	0.51	−0.26
IA8	381	1	5	2.41	1.062	0.471	−0.383
IA9	381	1	5	2.36	1.11	0.53	−0.421
IA10	381	1	5	2.38	1.033	0.495	−0.235
IA11	381	1	5	2.37	1.069	0.757	0.207
IA12	381	1	5	2.37	1.118	0.512	−0.388
IA13	381	1	5	2.46	1.069	0.522	−0.333
IA14	381	1	5	2.36	1.078	0.482	−0.55
IA15	381	1	5	2.41	1.046	0.429	−0.361
IA16	381	1	5	2.41	1.021	0.411	−0.354
IA17	381	1	5	2.37	1.032	0.518	−0.2
IA18	381	1	5	2.32	1.065	0.582	−0.295
IA19	381	1	5	2.39	1.076	0.521	−0.267
IA20	381	1	5	2.41	1.056	0.435	−0.36
IA21	381	1	5	2.37	1.07	0.522	−0.312
IA22	381	1	5	2.36	1.088	0.604	−0.213
IA23	381	1	5	2.37	1.06	0.391	−0.572
IA24	381	1	5	2.4	1.097	0.463	−0.466

### Sampling and data collection

The study adopted random sampling approach. Vocational students from four vocational colleges of Hebei province in China were invited in the survey [64]. Hebei Province is best for vocational education because of its strategic position near Beijing and Tianjin, government support, and emerging tech ecosystem. In addition, the Hebei provides numerous specialized affordable programs with easy access to diverse markets and skilled labor, advancing a convincing entrepreneurial environment [65].

In order to collect data, numerous approaches were adopted including phone calls, in person, website, etc. Vocational students enrolled in the four public colleges of China namely, Hebei institute of mechanical and electrical technology, Hebei vocational university of technology and engineering, Shi Jia Zhuang vocational and technical college, and Han Dan vocational and Technical College (total population 50000) [66]. Privacy of respondent personal information were also ensured. According to Cochran’s (1977) sample size formula, the minimum sample size for this study was 267 for a population of 50,000 with a 95% confidence level and a 6% margin of error. Out of 720 distributed surveys, 445 were obtained with a response rate of 61.8%. After screening, 64 data were excluded due to missing. For this study, sample size of 381 valid response were accepted, which is deemed adequate sample size and much larger than minimum threshold sample size (i.e., 267). In addition, numerous similar studies considered above 300 sample size is adequate for SEM on AMOS (Krejcie and Morgan, 1970; Israel, 1992; Wu et al., 2020).

Of the respondents in the study (see in the Table 1) 51.97% were female and 48.03% were male. These respondents were divided into different age groups, with more than half (52.23%) aged 19–20 years. Regarding grade distribution, most of the respondents were in their second year, accounting for 41.21%, while third-year students and above made up 40.68%. In terms of school distribution, Chongqing Vocational College of Economy and Trade accounted for the largest proportion of respondents, with 138 participants representing 36.22% of the total. Among the sample, 32.55% of the respondents majored in Accounting. From the perspective of student origin, the majority of respondents came from rural areas, accounting for 59.58%, while 40.42% were from urban areas. Concerning internship timing, 42.26% of the respondents reported internship periods in January and February, while 38.85% indicated an internship duration of five months or more.

**Table 1:** Demographic and background characteristics of respondents.

Frequency				
Items	Categories	N	Percent (%)	Cumulative Percent (%)
Gender	Male	183	48.03	48.03
	Female	198	51.97	100
Age	19-20	199	52.23	52.23
	21-22	108	28.35	80.58
	Above 23	74	19.42	100
Grade	First grade	69	18.11	18.11
	Second grade	157	41.21	59.32
	Third grade and above	155	40.68	100
School Name	Hebei Institute of Mechanical and Electrical Technology	66	17.32	17.32
	Hebei Vocational University of Technology and Engineering	138	36.22	53.54
	Shijiazhuang Vocational and Technical College	89	23.36	76.9
	Handan Vocational and Technical College	88	23.1	100
Professional Name	Accounting	124	32.55	32.55
	Business administration	59	15.49	48.03
	Auditing & Finance	40	10.5	58.53
	Marketing	76	19.95	78.48
	others	82	21.52	100
Student origin	Rural areas	227	59.58	59.58
	Urban areas	154	40.42	100
Duration of internship (months)	1-2 months	161	42.26	42.26
	3-4 months	72	18.9	61.15
	5 or above	148	38.85	100
Total		381	100	100

### Data analysis

A series with sequence of statistical analysis were applied including normality test, exploratory factor analysis (EFA), reliability test, correlation, confirmatory factor analysis (CFA), and Structural Equation Modeling (SEM) to investigate the associations among the constructs. SPSS and AMOS software were used to analyze data.

## Results

### Normality test

The normality of the items in this study was assessed using skewness and kurtosis values. Descriptive statistics for the variables are presented in Tables 2–4. The results indicate that the absolute values of skewness are below 3 and the absolute values of kurtosis are below 10, confirming that the data conforms to a basic normal distribution. Among the 20 items measuring the Big Five Personality Traits (Table 2), C2 recorded the lowest mean score, while ES4 recorded the highest. All variables had mean scores above the midpoint of 3 on a 5-point Likert scale, where 1 indicates “strongly disagree” and 5 indicates “strongly agree.” This suggests a general trend of agreement among respondents.

**Table 2:** Normality test of big five personality traits.

Variable name	Sample size	Minimum value	Maximum value	Mean value	Standard deviation	skewness	kurtosis
A1	381	1	5	2.38	1.017	0.839	0.334
A2	381	1	5	2.31	0.999	0.724	0.152
A3	381	1	5	2.5	1.048	0.534	−0.343
A4	381	1	5	2.47	1.024	0.606	−0.234
C1	381	1	5	2.21	0.994	0.758	0.133
C2	381	1	5	2.15	0.929	0.793	0.498
C3	381	1	5	2.22	1.064	0.85	0.154
C4	381	1	5	2.26	0.975	0.761	0.199
Ext1	381	1	5	2.53	1.012	0.24	−0.695
Ext2	381	1	5	2.53	1.012	0.3	−0.574
Ext3	381	1	5	2.55	1.089	0.459	−0.653
Ext4	381	1	5	2.52	1.033	0.394	−0.609
O1	381	1	5	2.32	1.095	0.737	−0.121
O2	381	1	5	2.48	1.08	0.506	−0.466
O3	381	1	5	2.4	0.986	0.516	−0.166
O4	381	1	5	2.6	1.104	0.343	−0.753
ES1	381	1	5	2.56	1.018	0.327	−0.655
ES2	381	1	5	2.61	1.047	0.324	−0.615
ES3	381	1	5	2.6	1.093	0.349	−0.782
ES4	381	1	5	2.82	1.06	0.026	−0.887

**Table 4:** Normality test of employability.

Variable name	Sample size	Minimum value	Maximum value	Mean value	Standard deviation	skewness	kurtosis
SS1	381	1	5	2.45	1.076	0.489	−0.413
SS2	381	1	5	2.43	1.13	0.526	−0.485
SS3	381	1	5	2.42	1.089	0.427	−0.55
SS4	381	1	5	2.4	1.048	0.359	−0.556
SS5	381	1	5	2.39	1.106	0.42	−0.684
SS6	381	1	5	2.42	1.07	0.411	−0.551
SS7	381	1	5	2.38	1.088	0.526	−0.41
SS8	381	1	5	2.41	1.062	0.484	−0.328
GBR1	381	1	5	2.62	1.028	0.341	−0.717
GBR2	381	1	5	2.53	1.032	0.431	−0.464
GBR3	381	1	5	2.57	1.092	0.485	−0.532
GBR4	381	1	5	2.51	1.065	0.435	−0.557
GBR5	381	1	5	2.59	1.057	0.359	−0.636
GBR6	381	1	5	2.64	1.216	0.46	−0.814
AA1	381	1	5	2.7	1.098	0.278	−0.723
AA2	381	1	5	2.7	1.088	0.271	−0.71
AA3	381	1	5	2.61	1.106	0.239	−0.818
AA4	381	1	5	2.68	1.223	0.34	−0.968
AA5	381	1	5	2.74	1.125	0.224	−0.797
AA6	381	1	5	2.67	1.093	0.339	−0.67
CK1	381	1	5	2.49	1.058	0.568	−0.258
CK2	381	1	5	2.5	1.058	0.479	−0.287
CK3	381	1	5	2.59	1.031	0.342	−0.559
CK4	381	1	5	2.54	1.122	0.443	−0.666
STA1	381	1	5	2.65	1.098	0.276	−0.796
STA2	381	1	5	2.7	1.11	0.316	−0.684
STA3	381	1	5	2.7	1.188	0.219	−0.907
STA4	381	1	5	2.57	1.061	0.422	−0.536
STA5	381	1	5	2.59	1.192	0.474	−0.765

For the Internship Attitude scale (Table 3), the mean scores ranged between 2.32 and 2.46, with IA13 achieving the highest mean. Similarly, for the Employability scale (Table 4), the mean scores were consistently above 2.39, with AA5 recording the highest mean score of 2.74. The results demonstrate acceptable normality and support the reliability of the data for subsequent analyses. Overall, the first-order variables across all scales reflect moderate to high levels of agreement, providing a robust foundation for further statistical modeling.

### Structural Equation Modeling (SEM)

Structural Equation Modeling (SEM) using AMOS 27 software was applied to analyze the relationships among the Big Five personality traits, internship attitudes, and students’ persistence intentions. The model included 45 free parameters and had a total of 7575 degrees of freedom (df = 7575). The SEM model met the required fit criteria, with CMIN/DF = 1.221, RMR = 0.043, and RMSEA = 0.024, indicating an excellent model fit. While GFI and NFI values were slightly below the ideal threshold of 0.9 (GFI = 0.827, NFI = 0.840), other critical fit indices, such as IFI = 0.967, TLI = 0.965, and CFI = 0.966, were well above the recommended threshold of 0.9. These results confirm that the structural model achieved an acceptable overall fit, supporting its validity and reliability (see Table 8).

**Table 8:** Model fit of SEM structural model.

Index	CMIN/DF	RMSEA	GFI	IFI	TLI	CFI	P
Judgment criteria	<3	<0.08	>0.9	>0.9	>0.9	>0.9	<0.05
Measurement result	1.221	0.024	0.827	0.967	0.965	0.966	0.000

### Exploratory factor analysis and reliability test

To assess the internal consistency of the constructed second-order latent constructs, a reliability test was conducted on the quantitative data [67]. Constructs with a Cronbach’s alpha value greater than 0.7 are considered to have good reliability (Hair et al., 2019). The Cronbach’s alpha values for the second-order latent constructs were Internship Attitude = 0.974 and Employability = 0.931, both exceeding 0.9, indicating very strong internal reliability. A high Cronbach’s alpha value (greater than 0.900) reflects strong internal consistency, demonstrating that the survey items are highly correlated with their respective constructs. This further confirms the alignment between first-order and second-order constructs, highlighting the robust measurement of the theoretical framework [68].

In addition, the Big Five Personality Traits, as an independent variable, had a Cronbach’s alpha value of 0.729, meeting the standard for good reliability (greater than 0.7). This indicates that the related items reliably capture the core characteristics of the Big Five Personality Traits. Overall, the results of the EFA and reliability tests support the theoretical foundation and measurement validity of all constructs. Details of the EFA, reliability tests, and the construction of these dimensions are presented in Table 5.

**Table 5:** Summary of latent and observed variables, EFA and reliability tests.

Latent Variables	Observed Variables	Items	Factor Loading	Cronbach Alpha
		KMO = .874		
Big Five Personality Trait	Agreeableness 1	During my internship, I sympathize with others’ feelings.	0.673	0.729
	Agreeableness 2	During my internship, I show interest in other people’s problems at work	0.799	
	Agreeableness 3	During my internship, I feel others’emotions.	0.691	
	Agreeableness 4	During my internship, I care about others at work.	0.725	
	Conscientiousness1	During my internship, I get my tasks done right away	0.716	
	Conscientiousness2	I am careful to put things back in their proper place at work.	0.697	
	Conscientiousness3	During my internship, I like order.	0.808	
	Conscientiousness4	During my internship, I am always prepared at work.	0.775	
	Extraversion1	During my internship, I feel comfortable around people.	0.664	
	Extraversion2	I make friends easily during my internship.	0.681	
	Extraversion3	During my internship, I am skilled in handling social situations.	0.834	
	Extraversion4	During my internship, I talk a lot at internship.	0.671	
	Openness1	During my internship, I enjoyed hearing different ideas.	0.758	
	Openness2	During my internship, I have a vivid imagination at work.	0.721	
	Openness3	During my internship, I enjoy thinking about things	0.713	
	Openness4	During my internship, I enjoy philosophical discussions at work.	0.712	
	Emotional stability1	During my internship, I am not easily bothered by things at work.	0.841	
	Emotional stability2	During my internship, I am relaxed most of the time.	0.721	
	Emotional stability3	During my internship, I don’t get upset easily at work.	0.713	
	Emotional stability4	During my internship, I remain calm under pressure.	0.712	
Internship Attitude	Internship Attitude1	I know how to reach out to the internship director for resources	0.783	0.974
	Internship Attitude2	I feel confident approaching my academic advisor for internship information	0.78	
	Internship Attitude3	I first heard about the COB internship office	0.793	
	Internship Attitude4	I started seeking out internship information	0.787	
	Internship Attitude5	I believe the most appropriate time to receive internship information	0.791	
	Internship Attitude6	I realized I needed to complete an internship	0.778	
	Internship Attitude7	I am likely to plan my internship at least a semester in advance.	0.777	
	Internship Attitude8	I think receiving internship information during my freshman year is helpful.	0.764	
	Internship Attitude9	I complete my tasks on time during my internship.	0.787	
	Internship Attitude10	I put things back in their proper place during my internship.	0.765	
	Internship Attitude11	I enjoy maintaining order during my internship.	0.775	
	Internship Attitude12	I prepare well in advance for my tasks during my internship.	0.776	
	Internship Attitude13	I enjoy hearing different ideas during my internship.	0.783	
	Internship Attitude14	I have a vivid imagination during my internship.	0.761	
	Internship Attitude15	I enjoy thinking deeply about issues during my internship.	0.797	
	Internship Attitude16	I am willing to engage in philosophical discussions during my internship.	0.762	
	Internship Attitude17	I am not easily bothered by things during my internship.	0.791	
	Internship Attitude18	I remain calm under pressure during my internship.	0.776	
	Internship Attitude19	I proactively ask my mentor questions during my internship.	0.792	
	Internship Attitude20	I actively seek to connect with new colleagues during my internship.	0.801	
	Internship Attitude21	I take the initiative to seek feedback during my internship.	0.789	
	Internship Attitude22	I have opportunities to apply the knowledge I learned in class during my internship.	0.78	
	Internship Attitude23	I feel satisfied with the allocation of tasks during my internship.	0.778	
	Internship Attitude24	I learn many skills during my internship that I cannot acquire in class.	0.786	
Employability	Soft Skill (SS)	I can obtaining and organizing information from its various sources	0.783	0.931
		I can performing mathematical and statistical applications	0.799	
		I can indulging in self-learning	0.76	
		I can adapting to the business environment	0.766	
		I can considering the ethical values and professional attitudes	0.785	
		I can exercising the required leadership skills	0.787	
		I can engaging with others to confront and resolve conflicts	0.79	
		I can negotiating acceptable solutions in different professional circumstances	0.75	
	General business requirements (GBR)	I have a fair knowledge about corporate governance	0.691	
		I have a fair knowledge about economic concepts	0.696	
		I have a fair knowledge about financial management	0.749	
		I have a fair knowledge about information technology	0.706	
		I have a fair knowledge about operations management	0.703	
		I have general business knowledge	0.773	
	Audit and assurance (AA)	I have capable of planning audit Processes	0.714	
		I can prepare various audit programs	0.704	
		I have capable of doing professional responsibilities related to auditing	0.709	
		I have capable assessing risk and developing a planned response in audits	0.855	
		I can perform further audit procedures and obtaining evidence	0.741	
		I have capable of forming conclusions and reporting on audits	0.702	
	Conceptual knowledge (CK)	I have knowledge about the basic accounting concepts and principles	0.67	
		I have the necessary theoretical knowledge to practice the accounting profession	0.759	
		I have the required knowledge for measurement	0.717	
		I have knowledge about accounting of taxes/zakat	0.797	
	Special topics in accounting (STA)	I know about government accounting	0.73	
		I know about government accounting	0.759	
		I know about insurance accounting	0.783	
		I know about accounting information systems	0.738	
		I know about special accounting issues	0.813	

### Confirmatory factor analysis

**Fig 2:**
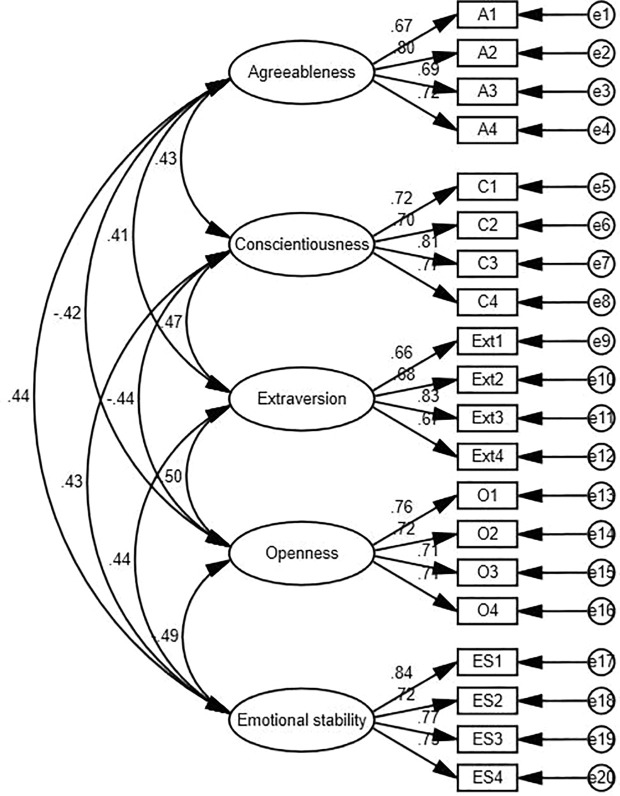
Confirmatory Factor Analysis (CFA) of Big Five Personality Traits.

**Fig 3:**
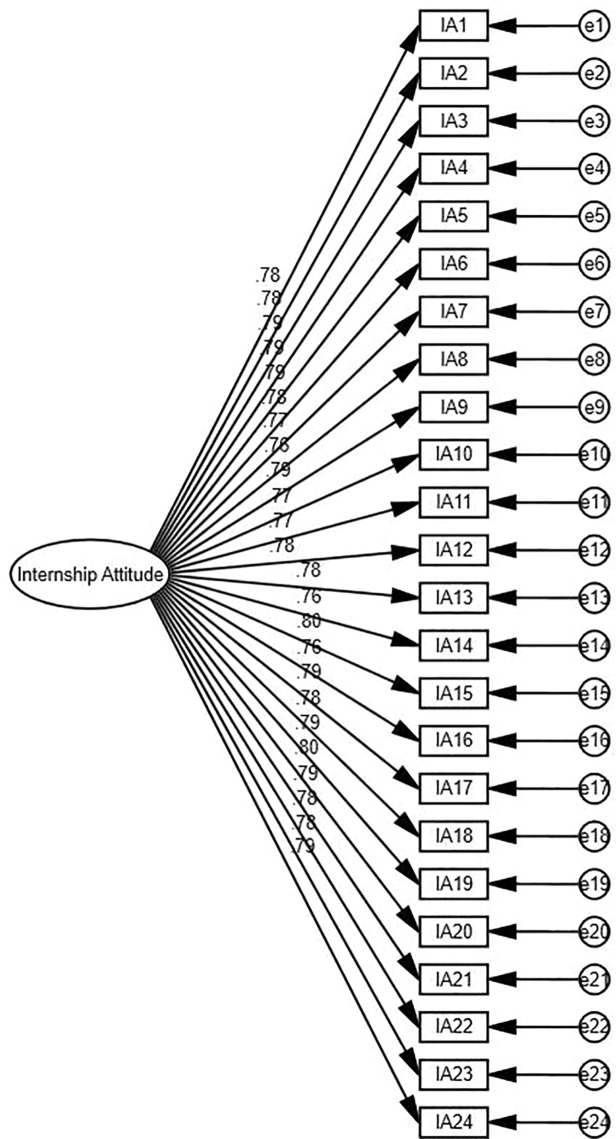
Confirmatory Factor Analysis (CFA) of Internship Attitude.

**Fig 4:**
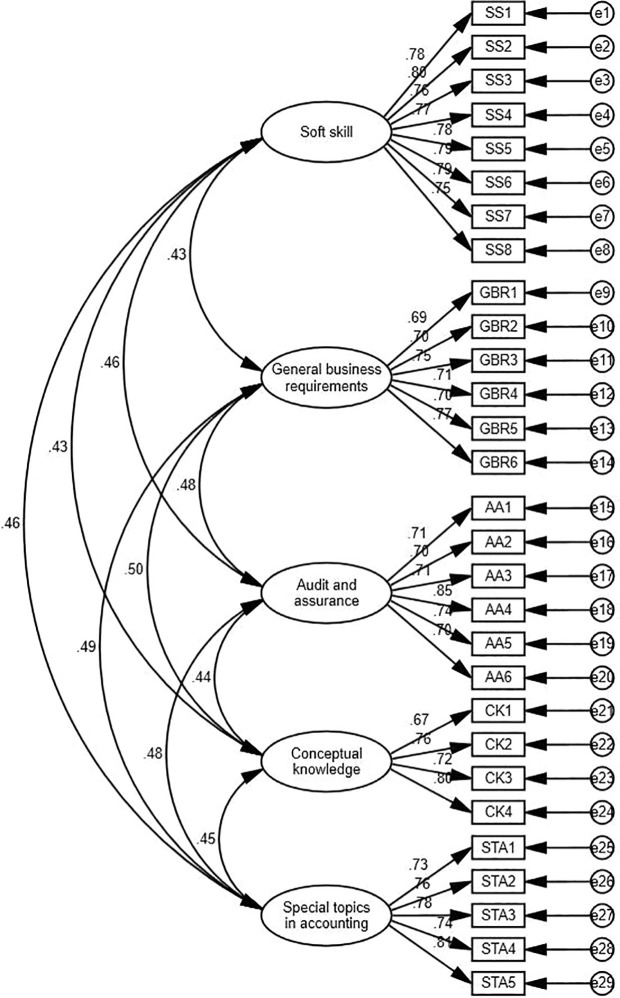
Confirmatory factor analysis of Employability.

According to Magistris and Gracia (2008), before doing hypothesis testing, confirmatory factor analysis (CFA) was performed to examine the validity of the variables (see Fig 2–4). This included establishment of measurement model using AMOS 27.0. It is commonly used to assess the primary conceptual model with a group of constructs for several hypotheses (Hair, 2009). It is a widely accepted technique used in business research to examine the measured constructs accurately denotes the underlying latent variable. CFA has been known as efficient and robust for testing validity (Wong 2013). To assess the reliability of the CFA model, numerous model fit indexes were checked, including the chi-square to degrees of freedom ratio (x2/df), comparative fit index (CFI), incremental fit index (IFI), normal fit index (NFI), goodness fir index (GFI), and root-mean-square error of approximation (RMSEA) (Kline et al., 2016). Wong (2013) indicated also that for marketing research, a significance level of 5%, a statistical power of 80%, and R2 values of at least.25 are considered typical.

In addition, another commonly reported statistic and a potential mechanism for accommodating large sample sizes may be to use the root mean square error of approximation (RMSEA) as a measure of goodness-of-fit in SEMs (Chen, Curran, Bollen, Kirby, & Paxton, 2008; and to measure the discrepancy per degree of freedom (Hu & Bentler, 1999). The AMOS 27 user’s guide suggests that “a value of the RMSEA of about 0.05 or less would indicate a close fit of the model in relation to the degrees of freedom” (Arbuckle, 2013). RMSEA values range from 0 to 1, with a smaller RMSEA value indicating better model fit. Acceptable model fit is indicated by an RMSEA value if recommendations for RMSEA cutoff points have been reduced considerably over the past couple of decades (Hu & Bentler, 1999).

The measurement models for the Big Five Personality Traits, Internship Attitude, and Employability were assessed using confirmatory factor analysis (CFA) in AMOS 27. Each model demonstrated excellent fit indices, confirming the constructs’ reliability and validity. For the Big Five Personality Traits, the fit indices were X^2^/df = 1.398, RMSEA = 0.032, CFI = 0.979, IFI = 0.979, and TLI = 0.975. For Internship Attitude, the indices were X^2^/df = 1.447, RMSEA = 0.034, CFI = 0.985, IFI = 0.985, and TLI = 0.983. Similarly, Employability showed excellent fit with X^2^/df = 1.350, RMSEA = 0.030, CFI = 0.978, IFI = 0.978, and TLI = 0.975. These results validate the measurement models and their suitability for structural modeling.

### Convergent validity

Combined reliability (Construct Reliability, CR value) and average variance extracted (AVE value) are used to evaluate convergent validity. The CR value, which represents the internal consistency reliability of constructs, is calculated based on factor loadings and is typically greater than 0.7. The AVE value, representing the average variance explained by a latent variable across its observed variables, is usually greater than 0.5. CR is calculated as the sum of squared factor loadings divided by the sum of squared factor loadings plus error variances. Higher CR values indicate stronger internal consistency.

Similarly, the AVE value is calculated as the sum of the squared factor loadings divided by the total number of items, representing the latent variable’s ability to explain its indicators. Higher AVE values indicate a stronger explanatory power of the latent variable, leading to better convergent validity. As shown in Table 5 and Table 6, all AVE values exceed 0.7, confirming strong convergent validity in this study. For example, in Table 5, “Soft Skill” has an AVE square root of 0.777, which exceeds its correlations with other constructs (ranging from 0.385 to 0.426). This demonstrates that the data have robust aggregation (convergence) validity.

**Table 6:** Convergent and discriminant validity.

Distinguishing validity: Pearson correlation and AVE square root values					
	Soft skill	General business requirements	Audit and assurance	Conceptual knowledge	Special topics in accounting
Soft skill	**0.777**				
General business requirements	0.385	**0.720**			
Audit and assurance	0.426	0.426	**0.740**		
Conceptual knowledge	0.382	0.424	0.394	**0.737**	
Special topics in accounting	0.411	0.434	0.433	0.388	**0.765**

Note: Diagonal black bold numbers are AVE square root values.

### Discriminant validity

The bold numbers in Table 4 and Table 5 represent the AVE square root values. The AVE square root values for Internship Attitude and Employability exceed the maximum absolute value of the correlation coefficients between constructs, confirming good discriminant validity. For example, the AVE square root value for Internship Attitude is 0.881, which is higher than the correlation coefficients between Internship Attitude and other constructs (ranging from 0.264 to 0.343). Similarly, the AVE square root value for Employability is greater than its correlations with other factors. These results indicate that both constructs are distinct from one another and from other latent variables. The findings for convergent validity (AVE) and discriminant validity are summarized in Table 6 and Table 7.

**Table 7:** Convergent and discriminant validity.

Pearson correlation							
	Agreeableness	Conscientiousness	Extraversion	Openness	Emotional stability	Internship Attitude	Employability
Agreeableness	1						
Conscientiousness	0.373^**^	1					
Extraversion	0.348^**^	0.387^**^	1				
Openness	−0.353^**^	−0.368^**^	−0.414^**^	1			
Emotional stability	0.377^**^	0.359^**^	0.378^**^	−0.421^**^	1		
Internship Attitude	0.409^**^	0.391^**^	0.390^**^	−0.395^**^	0.405^**^	1	
Employability	0.400^**^	0.411^**^	0.425^**^	−0.409^**^	0.414^**^	0.456^**^	1

* p < 0.05

** p < 0.01.

### Structural Equation Modeling (SEM)

Structural Equation Modeling (SEM) is a multivariate statistical technique used to examine the associations between exogenous and endogenous constructs in a conceptual model. It is extensively applied in management and business research to evaluate theoretical constructs and their interassociations. SEM involves two primary components: the measurement model and the structural model. This technique is widely recognized in management sciences for its ability to test complex associations among observed and latent variables across multiple levels [69].

In the current study, SEM was conducted to examine the associations among the Big Five Personality Traits, Internship Attitude, and Employability as second-order latent constructs. Fig 3 illustrates the SEM analysis, showing the standardized path coefficients and their significance levels. The research model posits that the Big Five Personality Traits positively influence Internship Attitude, which in turn positively influences Employability. The associations among these constructs, as depicted in the structural model, provide insights into the underlying mechanisms.

**Fig 5:**
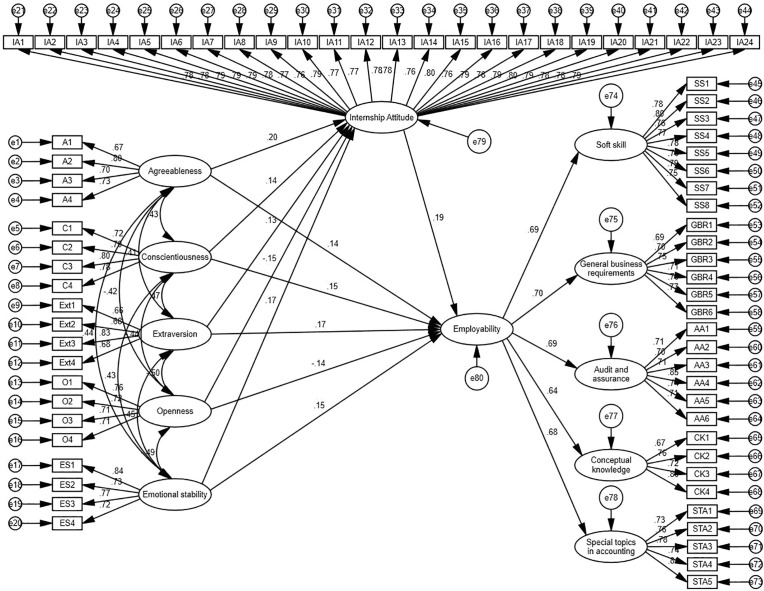
SEM structural model.

The SEM model was evaluated using several fit indices, including X^2^/df, RMSEA, CFI, IFI, and TLI. A good-fitting model is indicated by NFI, GFI, CFI, IFI, and TLI values close to or greater than 0.9. In this study, the SEM model met at least four threshold values of fit indices, ensuring its adequacy and reliability. Based on the hypothesized research model, a structural model was developed and analyzed (see Fig 5), providing robust evidence for the associations among the key constructs

The SEM results demonstrated that the model met the required fit criteria, with CMIN/DF = 1.221, RMR = 0.043, and RMSEA = 0.024, indicating an excellent model fit. While GFI and NFI values were slightly below the ideal threshold of 0.9 (GFI = 0.827, NFI = 0.840), other critical fit indices, such as IFI = 0.967, TLI = 0.965, and CFI = 0.966, were well above the recommended threshold of 0.9. These results confirm that the structural model achieved an acceptable overall fit, supporting its validity and reliability (see Table 8).

The maximum likelihood estimates of the final SEM model are presented in Table 9. The results show that the five dimensions of the Big Five Personality Traits (Agreeableness, Conscientiousness, Extraversion, Openness, and Emotional Stability) all have significant positive effects on Internship Attitude (e.g., Agreeableness = 0.134, p = 0.000; Conscientiousness = 0.142, p = 0.000; Extraversion = 0.157, p = 0.000; Openness = 0.131, p = 0.000; Emotional Stability = 0.139, p = 0.000). Additionally, Internship Attitude has a significant positive effect on Employability (β = 0.192, p = 0.000). These results indicate that the Big Five Personality Traits indirectly influence Employability through the mediating role of Internship Attitude, validating the hypothesized associations.

**Table 9:** Standard estimated likelihood of SEM model.

Path			Non-standard load factor	S.E.	C.R.	P	Standardized load coefficient
Internship Attitude	<---	Agreeableness	0.255	0.078	3.275	0.001	0.203
Internship Attitude	<---	Conscientiousness	0.169	0.074	2.275	0.023	0.14
Internship Attitude	<---	Extraversion	0.163	0.083	1.974	0.048	0.127
Internship Attitude	<---	Openness	−0.155	0.068	−2.277	0.023	−0.149
Internship Attitude	<---	Emotional stability	0.166	0.062	2.681	0.007	0.166
Employability	<---	Agreeableness	0.12	0.058	2.068	0.039	0.141
Employability	<---	Conscientiousness	0.123	0.055	2.215	0.027	0.151
Employability	<---	Extraversion	0.144	0.062	2.337	0.019	0.167
Employability	<---	Openness	−0.101	0.051	−1.998	0.046	−0.145
Employability	<---	Emotional stability	0.101	0.046	2.201	0.028	0.15
Employability	<---	Internship Attitude	0.126	0.042	3.012	0.003	0.188

The final SEM model and standard estimate loadings confirmed the hypothesized associations among for vocational students in China (see Table 10).

**Table 10:** Hypotheses testing.

	Hypothesis	Result
**H1**	There is a significant positive association between extraversion and employability.	Accepted
**H2**	There is a significant positive association between agreeableness and Employability.	Accepted
**H3**	There is a significant positive association between conscientiousness and Employability.	Accepted
**H4**	There is a significant negative association between neuroticism and Employability.	Accepted
**H5**	There is a significant positive association between openness to experience and Employability.	Accepted
**H6**	There is a significant negative association between extroversion and internship attitude.	Accepted
**H7**	There is a significant positive association between agreeableness and internship attitude.	Accepted
**H8**	There is a significant positive association between conscientiousness and internship attitude.	Accepted
**H9**	There is a significant negative association between neuroticism and internship attitude.	Accepted
**H10**	There is a significant negative association between openness to experience and internship attitude.	Accepted
**H11**	There is a significant positive association between internship attitude and employability.	Accepted
**H12**	Internship attitude mediates the association between extraversion and employability.	Accepted
**H13**	Internship attitude mediates the association between agreeableness and internship intention.	Accepted
**H14**	Internship attitude mediates the association between conscientiousness and internship intention.	Accepted
**H15**	Internship attitude mediates the association between neuroticism and internship intention.	Accepted
**H16**	Internship attitude mediates the association between openness to experience and continued internship intention.	Accepted

## Discussion and conclusion

### Discussion of research findings by objectives

This study provides critical insights into how the Big Five Personality Traits influence employability among vocational college students, addressing the first research objective. Conscientiousness, extraversion, and agreeableness emerged as the most significant positive traits, consistent with McCrae and Costa’s (1997) Five-Factor Model. Neuroticism showed a negative impact, confirming its detrimental role in stress management and adaptability. These findings align with Zhang et al. [70] research on job performance. Additionally, openness to experience exhibited a complex association, indicating its effects may vary depending on the specific cultural context of vocational education [71]. For the second research objective, this study explored the mediating role of internship attitudes, highlighting their critical function in linking personality traits with employability. Structural Equation Modeling (SEM) results confirmed that positive internship attitudes significantly enhance the impact of conscientiousness and agreeableness while mitigating the negative effects of neuroticism. These findings extend the discussion by Al-Swidi et al. [2] on the moderating effects of internship attitudes, demonstrating that fostering positive attitudes is essential for improving employability. The third objective focused on the association between personality traits and internship attitudes [4]. The results indicated that traits such as extraversion and agreeableness strongly predict proactive engagement and harmonious interactions during internships, whereas neuroticism undermines performance due to emotional instability. These findings not only validate Ng and Feldman’s (2009) research but also provide empirical support for theoretical discussions in vocational education within the Chinese context.

### Research significance

This study holds significant theoretical and practical implications. Theoretically, it constructs a systematic framework by integrating the Big Five Personality Traits and internship attitudes, enriching the understanding of employability. Unlike prior research that primarily focused on the direct effects of personality traits, this study highlights the mediating role of internship attitudes, offering a more nuanced perspective. The framework not only validates existing theories but also extends the understanding of vocational education by addressing cultural and contextual differences.

Practically, the findings provide valuable guidance for vocational education policies and internship program design. Educational institutions can use these insights to develop personality-based internship programs, tailoring support and mentorship to enhance student engagement and attitudes. Policymakers can prioritize supportive internship initiatives to better align educational outcomes with labor market demands. These strategies can significantly improve the employability of vocational students while enhancing the overall competitiveness and societal recognition of vocational education.

### Research limitations

Despite its contributions, this study has certain limitations. First, reliance on self-reported data introduces potential biases, such as social desirability effects. Second, the sample is limited to vocational students in Hebei Province. This geographical limitation restricts the generalizability of the findings to other regions or cultural contexts. The cultural background and educational environment in Hebei Province may have unique characteristics that influence the associations between personality traits, internship attitudes, and employability. Therefore, caution should be exercised when applying these findings to other regions. Future research should consider expanding the sample to include students from diverse regions and cultural contexts to enhance the generalizability of the results. Third, the cross-sectional design of this study limits the ability to establish causal associations between the variables. While Structural Equation Modeling (SEM) provides valuable insights into the correlations among the Big Five Personality Traits, internship attitudes, and employability, it cannot determine causality based on cross-sectional data. The findings should be interpreted as associations rather than causal effects. Accordingly, readers should interpret the structural paths as indicative of associations rather than directional or causal effects. Future research employing longitudinal or experimental designs would be better suited to uncover potential causal mechanisms and dynamic interactions among these variables.Future research should consider longitudinal designs to better understand the dynamic associations and potential causal pathways among these variables over time. Future research could adopt longitudinal designs to uncover the evolving associations among personality traits, internship attitudes, and employability.

### Future research directions

Future research could further expand on the findings in several ways. First, exploring alternative personality frameworks, such as the HEXACO model, may uncover additional predictors of employability. Second, longitudinal studies could provide deeper insights into how personality traits and internship attitudes evolve and interact over time. Third, extending the scope to include diverse educational systems and cultural contexts could validate the generalizability and applicability of the proposed framework. Lastly, the influence of external factors, such as organizational culture and mentor support, on internship attitudes and employability warrants further investigation to provide a more holistic understanding.

## Conclusion

This study underscores the significant impact of the Big Five Personality Traits on the employability of vocational college students and highlights the mediating role of internship attitudes. Through empirical analysis, the study offers an integrated framework connecting personality traits and internship attitudes, providing a theoretical and practical foundation for enhancing vocational education outcomes. The findings emphasize the importance of designing tailored internship programs and providing proactive mentorship to foster positive attitudes and maximize employability. As labor market demands continue to evolve, these insights can guide educational reforms and policy-making to help vocational college students achieve success in an increasingly competitive global economy.

## Supporting information

S1 File Main Dataset. This file contains the anonymized minimum dataset necessary to replicate the study findings.
(XLSX)
